# Bovine CD14 gene characterization and relationship between polymorphisms and surface expression on monocytes and polymorphonuclear neutrophils

**DOI:** 10.1186/1471-2156-9-50

**Published:** 2008-08-08

**Authors:** Eveline M Ibeagha-Awemu, Jai-Wei Lee, Aloysius E Ibeagha, Xin Zhao

**Affiliations:** 1 Department of Animal Science, McGill University, Ste-Anne-de-Bellevue, Quebec H9X 3V9, Canada; 2 Department of Tropical Agriculture and International Cooperation, National Pingtung University of Science and Technology, Neipu, Pingtung 912, Taiwan

## Abstract

**Background:**

CD14 is an important player in host innate immunity in that it confers lipopolysaccharide sensitivity to cell types like neutrophils, monocytes and macrophages. The study was aimed at characterizing the CD14 gene of cattle for sequence variations and to determine the effect of variations on the expression of the protein on the surfaces of monocytes and neutrophils in healthy dairy cows.

**Results:**

Five SNPs were identified: two within the coding regions (g.A1908G and g.A2318G, numbering is according to GenBank No. EU148609), one in the 5' (g.C1291T) and two in the 3' (g.A2601G and g.G2621T) untranslated regions. SNP 1908 changes amino acid 175 of the protein (p.Asn175Asp, numbering is according to GenBank No. ABV68569), while SNP 2318 involves a synonymous codon change. Coding region SNPs characterized three gene alleles *A *(GenBank No. EU148609), *A*_1 _(GenBank No. EU148610) and *B *(GenBank No. EU148611) and two deduced protein variants A (ABV68569 and ABV68570) and B (ABV68571). Protein variant A is more common in the breeds analyzed. All SNPs gave rise to 3 haplotypes for the breeds. SNP genotype 1908AG was significantly (P < 0.01) associated with a higher percentage of neutrophils expressing more CD14 molecules on their surfaces. The promoter region contains several transcription factor binding sites, including multiple AP-1 and SP1 sites and there is a high conservation of amino acid residues between the proteins of closely related species.

**Conclusion:**

The study has provided information on sequence variations within the CD14 gene and proteins of cattle. The SNP responsible for an amino acid exchange may play an important role in the expression of CD14 on the surfaces of neutrophils. Further observations involving a larger sample size are required to validate our findings. Our SNP and association analyses have provided baseline information that may be used at defining the role of CD14 in mediating bacterial infections. The computational analysis on the promoter and comparative analysis with other species has revealed regions of regulatory element motifs that may indicate important regulatory effects on the gene.

## Background

The bovine cluster of differentiation (CD) 14 is an important player in host innate immunity in that it mediates host defense against Gram-negative bacterial infections and also confers immunity against viral infections [1,2]. It is abundant (about 99,500 to 134,600) on the cell membrane of monocytes and to a lesser extent (about 1,900 to 4,400) on neutrophils (polymorphonuclear neutrophil leukocytes) [3,4]. Two forms exist, a membrane bound form (mCD14) and a soluble (sCD14) form [5]. sCD14 is known to confer lipopolysaccharide (LPS) sensitivity to cells lacking mCD14, including epithelial cells and endothelial cells [6,7]. Also, recombinant bovine sCD14 can sensitize mammary epithelial cells to low concentrations of LPS *in vivo *and *in vitro *thus indicating an important role of sCD14 in initiating host responses to Gram-negative bacterial infections [8,9]. During the periparturient period, individual variations have been noticed in the response of cows to Gram-negative and Gram-positive bacteria infections [10]. Therefore, sequence variations of the CD14 gene may play important roles in the presentation of CD14 molecules and thus, LPS sensitivity.

The CD14 gene of cattle was initially cloned and sequenced by Ikeda et al. [11] and recently by the bovine genome project. It is mapped to BTA 7. A SNP of human CD14 promoter has been shown to influence the activities of the gene [12]. Baldini et al. [13] reported a SNP in the human CD14 gene promoter involving a C to T transition at position -159 (or -260 by [14]), and associated TT homozygotes with significantly higher levels of sCD14 and lower levels of IgE in children as compared to carriers of TC or CC. Reports of association of the -159 CD14 polymorphism with several human disease conditions have emerged [15-17]. Other authors however did not find any association between this polymorphism and several diseases [18,19]. Despite the importance of this protein and the effect of the promoter polymorphism in humans, there is no report of sequence variations of this gene in cattle and their possible effects on circulating CD14 levels and disease susceptibility.

The objectives of this study were therefore to: (1) investigate the CD14 gene of cattle for sequence variations; (2) determine the effect of variations on CD14 expression on the surfaces of monocytes and neutrophils; and (3) use bioinformatics tools to computationally characterize the promoter region. In this study we present information on sequence variations within the CD14 gene of Canadian Holstein and Jersey cows and a possible role of one SNP in influencing the surface expression of the antigen on the surfaces of neutrophils. Furthermore, identified conserved regions of regulatory element motifs may have important regulatory effects on the gene. The results may provide baseline information that may be used in candidate gene studies aimed at defining the role of CD14 in mediating bacterial infections.

## Results

### SNPs in the CD14 gene of Canadian Holsteins and Jersey cows

Comparison of the CD14 sequences of 106 Canadian Holsteins and 46 Jersey cows with published sequences (GenBank Nos. NW_001495367 and D84509) revealed a total of five SNPs including one in the 5' untranslated region (UTR) (g.C1291T, numbering is according to GenBank No. EU148609), two in the coding regions (g.A1908G and g.A2318G) and two in the 3' UTR (g.A2601G and g.G2621T) (Table 1). Four of the SNPs are transitional mutations while SNP 2621 involves the transversion of guanine to thymine. SNP 1908 is responsible for a non-synonymous codon change in amino acid 175 of the protein, from Asn (**a**ac) to Asp (**g**ac), while SNP 2318 results in a synonymous codon change without a change in amino acid 311 (Pro, cc**a **vs cc**g**) of the protein. The coding region SNPs characterizes three gene alleles *A *(A_1908_A_2318_) (GenBank No. EU148609), *A*_1 _(A_1908_G_2318_) (GenBank No. EU148610) and *B *(G_1908_G_2318_) (GenBank No. EU148611) and two deduced protein variants A (Asn175) (A_1908_A_2318_, GenBank No. ABV68569 or A_1908_G_2318_, GenBank No. ABV68570) and B (Asp175) (G_1908_G_2318_, GenBank No. ABV68571). Protein variant A is more common in the breeds analyzed with a frequency of 88.6% in Holsteins and fixed in Jerseys (Table 1). Within protein variant A, gene allele *A*_1 _occurred at a very high frequency (80.2%) in Jerseys. The non-coding SNPs occurred at about equal magnitudes in Holsteins while the frequencies of T_1291_, G_2601 _and T_2621 _were above 80% in Jerseys.

Comparison of deduced protein sequences with reported CD14 protein sequences for cattle (UniProt AAD32215 and UniProt NP_776433) revealed a further amino acid difference, 209Ser→Thr. Further comparisons revealed that amino acid 175Asn is conserved in cattle, buffalo (UniProtKB ABE68724), goat (UniProtKB ABE68725) and sheep (UniProtKB NP_001070677) while Asp is present at this position in cattle variant B.

**Table 1:** SNPs, gene alleles, protein variants, haplotypes and their frequencies in the analyzed breeds

Parameter	Holstein (n = 106)	Jersey (n = 43)
C1291T*		
*C*	0.658	0.198
*T*	0.342	0.802
A1908G		
*A*	0.886	1.000
*G*	0.114	-
A2318G		
*A*	0.658	0.198
*G*	0.342	0.802
A2601G		
*A*	0.658	0.198
*G*	0.342	0.802
G2621T		
*G*	0.658	0.198
*T*	0.342	0.802
Gene alleles (haplotypes)		
*A *(*CAAAG*)	0.658	0.198
*A*_1 _(*TAGGT*)	0.228	0.802
*B *(*TGGGT*)	0.114	-
Protein variants		
A or **175Asn	0.886	1.000
B or 175Asp	0.114	-

*SNP Numbers are according to GenBank No. EU148609

** Amino acid residue numbering is according to GenBank No. ABV68569

### Haplotye structure of the breeds

Considering the five SNPs identified and the genotype information of all individuals sequenced, the program PHASE V2.2.1 determined a total of four potential haplotype combinations (CAAAG-C_1291_A_1908_A_2318_A_2601_G_2621_, CGAAG, TGGGT, TAGGT) in the analyzed breeds. TGGGT was absent in Jersey while CGAAG was absent in both populations. The gene alleles *A1 *and *B *were observed from the sequencing data to be associated with SNPs T_1291_, G_2601 _and T_2621 _while allele *A *was associated with C_1291_, A_2601 _and G_2621 _therefore giving rise to three actual haplotypes (TAGGT, TGGGT and CAAAG) in the analyzed populations (Table 1). The frequency of the haplotype associated with allele *A *(CAAAG) was highest (65.8%) in Holsteins while the haplotype associated with allele *A*_1 _(TAGGT) was highest in Jerseys (80.2%) (Table 1).

### Effects of CD14 genotypes on the expression of CD14 on the surfaces of monocytes and neutrophils

**Figure 1:**
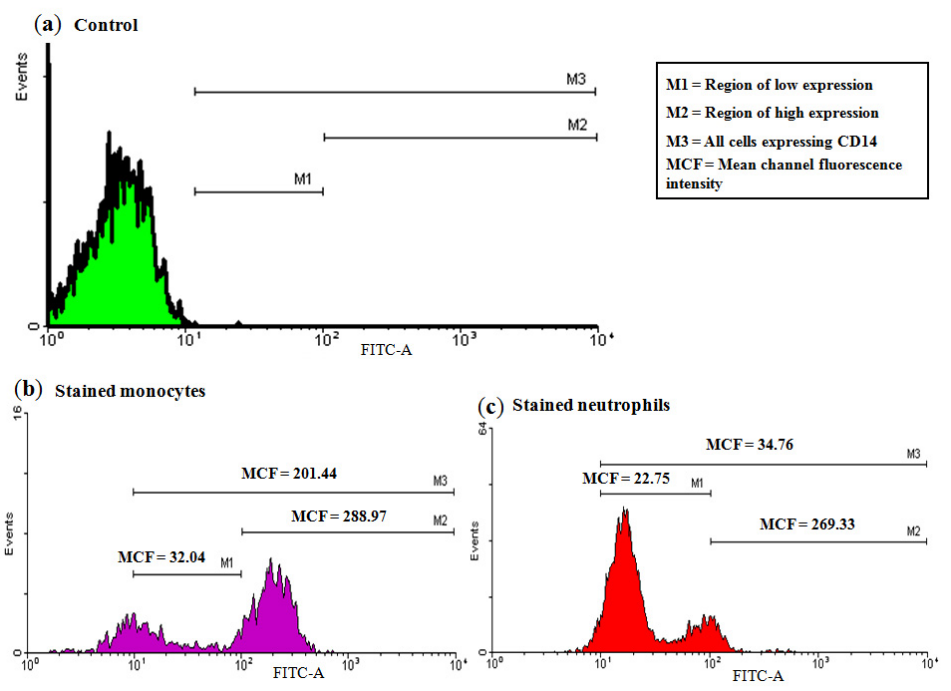
Flow cytometyric analyses of relationship of surface expression of CD14 on monocytes and neutrophils with CD14 genotypes. Cells were stained with fluorescein isothiocyanate (FITC)-labelled mouse anti human CD14 antibody and 20,000 events were gated and analyzed with Windows Multiple Document Interface for flow cytometry (WinMDI) software version 2.8. (a) Histogram showing a control sample that was not stained with antibody and occupies the Log10^0 ^to 10^1 ^region and known as the control zone. M1 (Log10^1 ^to 10^2^) is the zone of low expression or lower fluorescence zone indicating lower number of CD14 antigens on cells, M2 (Log10^2 ^and higher) is the zone of higher fluorescence emitted by a higher rate of absorption by more CD14 antigens on cells and M3 is the total area of expression. (b) Histogram showing stained monocytes with a higher percentage of cells in M2 and a higher overall MCF intensity of 201.44 as compared to 34.76 for polymorphonuclear neutrophils; (c) Histogram showing stained polymorphonuclear neutrophils with a higher percentage of cells in M1.

Whole blood from healthy Holstein cows (animals showing no outward symptoms of infection and farm record indicating milk somatic cell counts below 200,000 cells/ml) with different CD14 genotypes were incubated with fluorescein isothiocyanate (FITC)-labeled mouse anti-human CD14 antibody to determine the effects of genotypes on the expression of CD14 antigens on the surfaces of monocytes and neutrophils. The results are presented in Figure 1 and Table 2. In Figure 1, a higher percentage of gated monocyte cells were found in the LogFITC region labeled M2 (log10^2 ^and above) which indicates a higher fluorescence intensity coming from cells with the most CD14 antigens on their surfaces and termed the high expression region. On the other hand, more neutrophils were in the M1 or low expression region (Log10^1 ^to 10^2^). The mean channel fluorescence (MCF) intensities (for all cows) observed for the gated regions were M1 = 32.04 (range 16.67 – 65.18), M2 = 288.97 (149.85 – 566.47) and M3 = 201.44 (73.57 – 437.56) for monocytes and M1 = 22.76 (16.20 – 35.52), M2 = 267.33 (148.26 – 536.87) and M3 = 34.76 (19.49–60.83) for neutrophils. As presented, the M3 (M1 + M2) MCF intensity for monocyte was higher than for neutrophils. SNP A1908G that changes amino acid 175Asn to Asp, and thus protein A to B, is significantly (P < 0.01) associated with a higher number of neutrophils in the M2 or higher expression zone. 7.26% of gated neutrophils from cows of genotype 1908AG were found in M2 as compared to 4.36% from cows of genotype 1908AA (Table 2). In the M1 zone, the percentage of neutrophils from cows of both genotypes was the same. For monocytes, a higher number of total cells stained was observed for 1291CT (P < 0.05) and the other genotypes (2318AG, 2601AG and 2621GT) in perfect linkage disequilibrium with C1291T but this difference disappeared when Scheffe's adjustments was applied to means. A similar result was recorded for haplotypes.

**Table 2:** Effects of CD14 genotypes on its expression (in %) on the surfaces of monocytes and neutrophils in Holstein cows (n = 64)

Parameter	Genotypes	No.	Monocytes			Neutrophils		
		
			*Low	High	Total	Low	High	Total
**C1291T	CC	28	29.57 ± 1.54	57.00 ± 1.81	86.57^a ^± 0.96	73.25 ± 2.52	4.48 ± 0.62	77.74 ± 2.73
	CT	30	28.25 ± 1.48	61.26 ± 1.75	89.51^b ^± 0.93	76.21 ± 2.43	5.54 ± 0.60	81.75 ± 2.64
	TT	6	32.02 ± 3.32	58.94 ± 3.91	90.97^b ^± 2.07	81.57 ± 5.43	4.11 ± 1.34	85.68 ± 5.91
								
A1908G (Asn175Asp)	AA	51	29.10 ± 1.14	59.12 ± 1.36	88.22 ± 0.74	75.25 ± 1.88	4.36^A ^± 0.43	79.60 ± 2.03
	AG	13	29.49 ± 2.26	59.39 ± 2.70	88.88 ± 1.47	76.09 ± 3.72	7.26^B ^± 0.86	83.35 ± 4.03
								
Haplotypes	CAAAG, CAAAG	28	29.57 ± 1.56	57.00 ± 1.82	86.57^a ^± 0.97	73.25 ± 2.55	4.48^a ^± 0.59	77.74 ± 2.78
	CAAAG, TAGGT	20	27.75 ± 1.84	62.46 ± 2.16	90.21^b ^± 1.14	77.36 ± 3.01	4.45^a ^± 0.70	81.81 ± 3.28
	CAAAG, TGGGT	10	29.24 ± 2.60	58.85 ± 3.05	88.09^a ^± 1.61	73.90 ± 4.26	7.73^bc ^± 0.98	81.63 ± 4.65
	TAGGT, TGGGT	3	30.33 ± 4.75	61.18 ± 5.56	91.51^a ^± 2.95	83.41 ± 7.78	5.68^ab ^± 1.80	89.08 ± 8.48
	TAGGT, TAGGT	3	33.72 ± 4.75	56.72 ± 5.56	90.42^a ^± 2.95	79.73 ± 7.78	2.55^a ^± 1.80	82.28 ± 8.48

^a, b, c or A, B^Means for each parameter and within the same column differ significantly. a, b, c indicates significance (P < 0.05) with non adjusted means while A, B indicate significance with both non-adjusted means and when Scheffe's adjustments were applied to means (P < 0.01). SNPs 1291, 2318 and 2601 are in 100% linkage disequilibrium and possess the same information, like wise are gene alleles and haplotypes. Only values for SNP1291 and haplotypes have been represented in the table.

*Low indicates the percentage of cells in the region of Log10^1 ^to 10^2^, high > log10^2 ^and total, all cells stained.

**SNP numbering is according to GenBank No. EU148609 and amino acid residue numbering is according to GenBank No. ABV68569.

### Characterization of CD14 promoter and comparative analysis ofCD14 proteins

**Figure 2:**
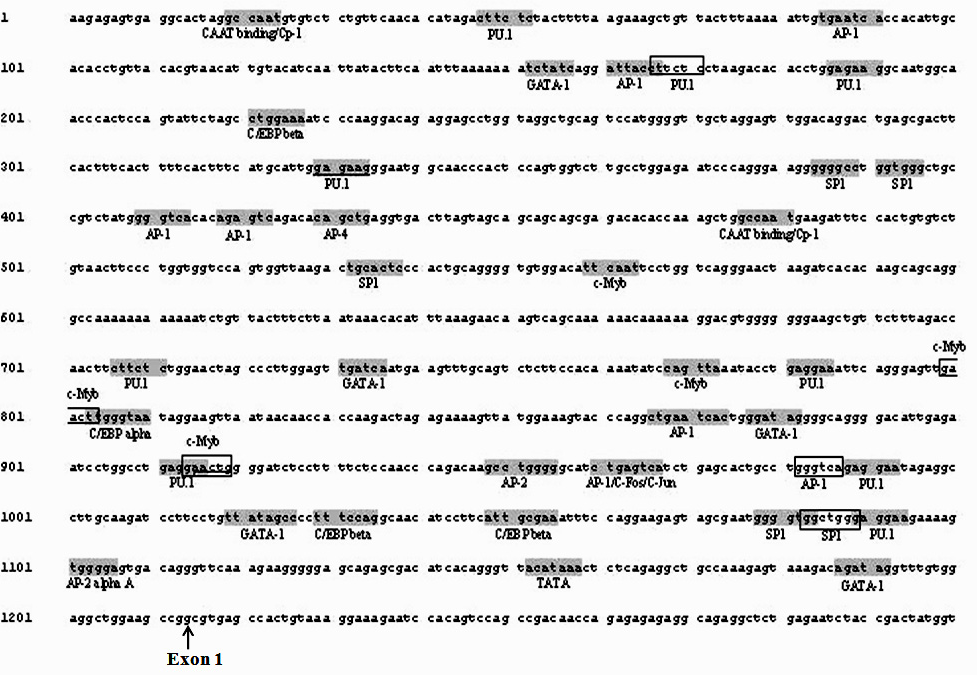
Putative transcription factor binding sites (with 100% match) on the bovine CD14 promoter (1213 bps). Recognition sequences are shaded. The rectangle is used where recognition sequences overlap. The arrow points to the first nucleotide of exon 1 at position 1214. Numbering is according to GenBank No. EU148609.

The CD14 sequence analyzed in this study is made up of 2630 bps with a 1213 bp promoter (Figure 2), two exons and one intron (GenBank No. EU148609). With the use of bioinformatics tools and published information in the literature, we were able to identify putative transcription factor binding sites (TFBSs) (with 100% match against searched data bases) on the bovine CD14 promoter. The putative TFBSs described here are, in particular, those already demonstrated to control the expression of the gene in human and rat. The putative motifs are shown in Figure 2 (boxed) and include amongst others 9 PU.1, 7AP-1, 5 SP1, 4 C/EBP, 3 c-Myb and 2 AP-2 sites.

**Figure 3:**
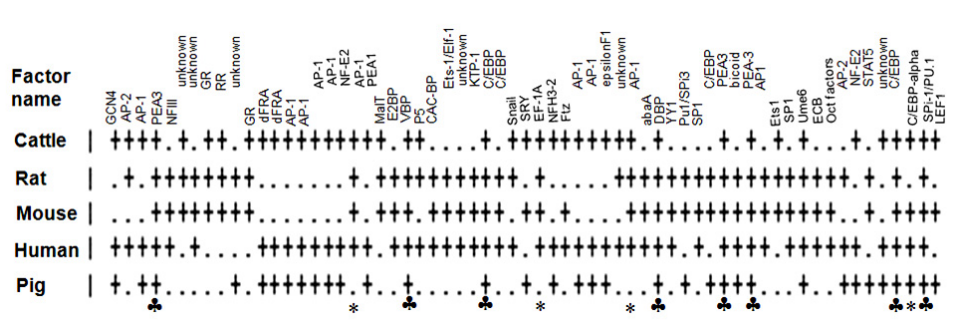
Conserved motifs of regulatory elements in the core promoter regions (about 500 bps) of cattle (GenBank No. EU148609), rat (GenBank No. AF087944), mouse (GenBank No. X13987), human (GenBank No. U00699) and pig (GenBank No. DQ079063). + indicates the presence of a motif and • its absence. ♣ indicates motifs that are conserved in all five species and * in four species including cattle. A maximum of three base pair mismatches was allowed.

We also searched for conserved regulatory motifs in the core promoters of the CD14 genes of different species (cattle, human, mouse, rat and pig) through a query in the NSITEM data base . The results indicated a total of 63 motifs of 50 regulatory elements (REs) that were conserved in the analyzed breeds with zero to 3 bp mismatches (Figure 3). Nine of the motifs representing 6 REs including C/EBP and Spi-1/PU.1 were conserved in all the species while 4 motifs of 3 REs including AP1 and C/EBP-alpha were conserved in 4 species including cattle (Figure 3). Furthermore, 60.30% (38) of the motifs were conserved between cattle and at least two other species. Sequence alignment of the same region revealed a perfect conservation, both in nucleotide number and orientation of the TATA box in cattle, human, mouse and rat (data not shown). The TATA box of pig differed from the others by only one bp mismatch.

**Figure 4:**
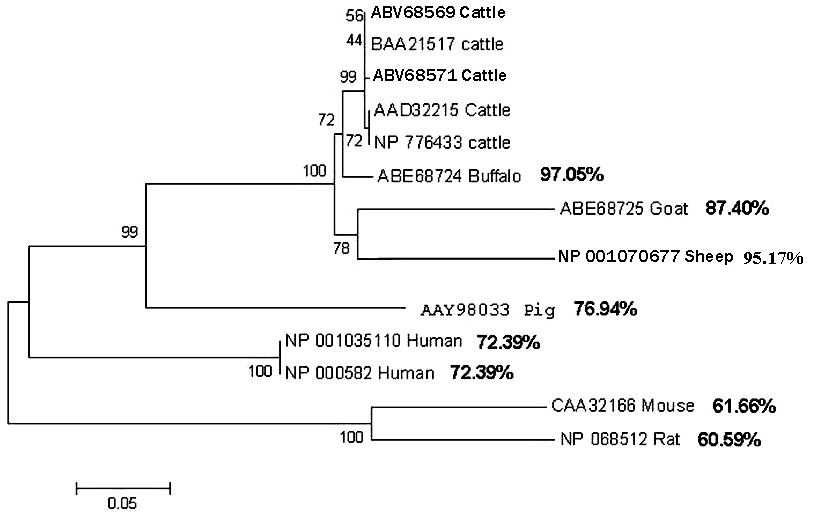
A Neighbor-joining dendrogram of the phylogenetic relations among the CD14 proteins of cattle, buffalo, goat, sheep, pig, human, mouse and rat. Species common names are preceded by their GenBank numbers. The degree of amino acid conservation between the bovine proteins and other species is represented in percentages. On the nodes are percent bootstrap values.

Further, we checked the degree of conservation between the CD14 proteins of cattle with those of different species. Protein sequences compared were those deduced in this work, variants A (ABV68569) and B (ABV68571), other published bovine sequences UniProt: BAA21517, AAD32215 and NP_776433, protein sequences of buffalo (ABE68724), goat (ABE68725), sheep (NP_001070677), pig (AAY98033), mouse (CAA32166), rat (NP_068512) and human variant 1 (NP_001035110) and 2 (NP_000582). The analysis revealed extensive conservations in the amino acid composition and structure. For the bovine proteins, the sequence of ABV68569 is the same as BAA21517 while AAD32215 and NP_776433 differed from ABV68569 by having amino acid 209 changed from Ser to Thr. This indicates the presence of a further CD14 protein variant in cattle here named C. Furthermore, the amino acids of the CD14 proteins of buffalo, sheep and goat shared high conservation rates of 97.05%, 95.17%, and 87.40% respectively, with the bovine ABV68569, followed by pig (76.94%) (Figure 4). The rate of amino acid conservation of bovine A variant was less with the human (72.39%), mouse (61.66%) and rat (60.59%) proteins. While bovine, buffalo, goat, sheep and pig proteins are made up of 373 amino acids, the human protein has two more, and the rat and mouse have one more and seven less animo acids, respectively. The signal peptide, made up of the first 20 amino acids, was highly conserved (only one amino acid difference, 14Ser to Pro) between the bovine and buffalo/goat/sheep proteins. The difference between cattle and the pig was 4 amino acids while being highly diverged with human, mouse and rat (7 to 12 amino acid differences). These relationships were further represented phylogenetically (Figure 4). As depicted in Figure 4, three main groups were evident; the rat and mouse proteins in group one (bootstrap value 100), the human proteins in another group (bootstrap value 100) while members of the Artiodactyla order (bovine, buffalo, goat, sheep) and pig formed a group of their own (bootstrap value 99). In the third group, the pig formed an outcrop of its own, while a closer relationship was visible between cattle and buffalo on the one hand (bootstrap value 72) and, goat and sheep on the other hand (bootstrap value 78).

## Discussion

We report here sequence variations of the bovine CD14 gene of Canadian Holstein and Jersey cows, through genomic DNA sequencing and computational analysis of the promoter. A gene is made up of both coding and non-coding regions which are all important in its expression and functionality. The complete description of a gene must therefore contain necessary information about the protein coding regions [20] and non-coding regions. The CD14 gene is an important component in host immunity [1,8] and detailed information on its structure and sequence variations as shown in this study may provide further insight into its mode of action.

The coding region SNPs in our study and comparative analysis of our sequences with published sequences show that the CD14 gene of cattle codes for three putative CD14 proteins-A (GenBank No. ABV68569 and ABV68570), B (GenBank No. ABV68571) and C (UniProt AAD32215 and UniProt NP_776433), with A and B described herein. The A variant, fixed in Jerseys and with a high frequency of 88.6% in Holsteins, may be the original wild type allele for the gene. Furthermore, the sequence of UniProt BAA21517 is similar to variant A and the haplotyes that contain the A variant SNPs are at the highest frequencies in the studied breeds. The other variants may therefore be the result of recent mutational events. Further three SNPs described in the 5' and 3'UTRs and one synonymous SNP in the coding region of the gene indicates a higher sequence variation for the gene in Canadian Holsteins than Jerseys.

The variations, both in the coding and non-coding regions of the gene may affect the surface expression of CD14 molecules on monocytes and neutrophils. Interestingly, the coding region SNP that gave rise to the B variant of the protein (g.A1908G or p.Asn175Asp) had the greatest effect by being associated with the highest number of neutrophils expressing more CD14 molecules on their surfaces. It is well known that, monocytes express more CD14 receptors on their surfaces, about 99,500 to 134,600 as compared to 1,900 to 4,400 for neutrophils (3). This difference was clearly shown by the pattern of expression depicted in Figure 1 whereby, more monocytes were recorded in the M2 gated zone (region of higher expression) and more neutrophils in the M1 zone (lower expression). These results suggest that, the characteristic B protein SNP or g.A1908G may play a role in cell surface expression of CD14 on neutrophils. Also, the SNP in the 5'UTR region could be important in influencing the expression of this receptor on monocytes. Our data was however based on a small sample size necessitating further verifications on a larger scale. Neutrophils form a major line of defense against bacterial infections and their effectiveness depends on their availability at the site of infections. For Gram-negative bacterial infections, the CD14 molecule confers LPS sensitivity to neutrophils [4], which is necessary to initiate host immune responses. The complex of TLR4, CD14 and myeloid differentiation protein 2, enhanced by the presence of LPS binding protein is crucial in LPS signaling; leading to the release of cytokines [21-23].

Even though no promoter polymorphism was detected in this study, a promoter polymorphism of the gene in human is a risk factor in several diseases [15-17]. The promoter region in bovine may probably be under strong purifying selection which may explain the lack of SNPs in this region. This is a positive factor considering the important role of the gene in mediating Gram-negative bacteria attack and the possible effect of the 1908 SNP on the abundance of the molecule on the surfaces of neutrophils. Determination of the roles of the individual SNPs and haplotypes on the activities of the gene under disease conditions will further shed more light on their biological significance.

Our analysis on promoter characterization indicates that part of the exon 1 reported by Ikeda et al. [11] constitutes the promoter. Since evolutionary pressures lead to the conservation of important non-protein-coding regulatory regions, including transcription factor binding sites (TFBs) across closely related species, identification of TFBs described in the CD14 genes of human [24] and rat [25] in the present study was expected. In particular, the perfect conservation of the TATA box, 9 motifs of 6 REs motifs across cattle, human, rat, mouse and pig and 4 other motifs across at least 4 of these species including cattle shows common regions in the core promoter that act together in the same biological context to control the expression of gene products and functions. In our study, up to 5 SP1 and 8 AP1 sites (with no bp mismatch) were identified which may indicate possible roles in controlling the expression of the gene as demonstrated in the rat and humans [24,25]. In the rat, Lui et al. [25] through mobility shift assays demonstrated that the SP1 and AP1 elements located respectively at positions -836 and -270 were required for basal promoter activity in liver cells. Also, Zhang et al. [24] showed that the SP1 transcription factor bound to three different regions of the human CD14 promoter and that a mutation of the major SP1 binding site decreased tissue specific promoter activity. One of the AP1 sites in our study, also shared by c-Fos and c-Jun (position 960–967, Figure 2), is similar to an AP1 site in rat promoter were JunD and Fra-2 proteins have been shown to bind [25]. This site is also thought to transactivate the basal expression of the gene [25]. This site in the mouse also plays a major role in the expression of the CD14 gene in macrophages [26]. Furthermore, three motifs of PEA3 interestingly were conserved in the promoters of all species studied. PEA3 belongs to the ETS transcription factor super family and is known to appear on the promoters of many cellular genes, including HER-2/neu [27] and CD226 antigen [28]. Conservation of C/EBP or CCAAT/enhancer binding proteins motifs in the studied species may be explained by their involvement in many aspects of cell growth. The high conservation of the amino acids of the proteins of bovine, buffalo, sheep and goat proteins was reflected in the tree of their phylogenetic relationships and is in line with other studies that found a high rate of conservation of genes and protein coding nucleotide positions between bovine, sheep and goat genes [29,30]. This further supports the fact that information from the sequencing of the bovine genome will greatly enhance studies in other very closely related species.

## Conclusion

Overall, this study provides information on sequence variations of the CD14 gene of Canadian Holstein and Jersey cows. The identified variations and association data have provided information that may shade more light on cell surface expression of CD14 by neutrophils, which are needed to control bacterial infections. Further data on the biological significance of the mutations is however necessary. Our computational analysis highlighted on the regulatory element motifs present in the promoter region of the gene. The comparative analysis with other species revealed conserved regions of regulatory element motifs that may have important regulatory effects on the gene.

## Methods

### Animals and genomic DNA extraction

Genomic DNA was extracted from the blood of 106 Canadian Holstein cows kept at the Howard Webster Centre-Macdonald Teaching Farm, McGill University and the milks of 46 Jersey cows enrolled in the Quebec Dairy Production Centre of Expertise program  using Nucleospin Blood Mini Kit (Macherey-Nagel Inc. Easton, PA) as described by the manufacturer. In the case of milk DNA isolation, the manufacturer's protocol was slightly modified. Milk samples were initially centrifuged at 13000 rpm for 15 min at 4°C to remove excess fat before proceeding with the manufacturer's protocol.

### PCR amplification and sequencing

Four primer pairs were designed with Invitrogen's OligoPerfect™ software (Invitrogen, Canada Inc., Burlington, ON, Canada) based on GenBank No. NW_001495367 and used to amplify overlapping regions covering the whole *Bos taurus *CD14 gene (Table 3). Invitrogen synthesized the primers.

**Table 3:** Primers used in the amplification of the whole bovine CD14 gene and other PCR conditions

Primer	*Primer sequence	MgCl_2 _concentration	Amplicon size (bp)
BoCD14.83330F	5'ATT ACC TTC TTC TGC ACC TCC A 3'	2.5 mM	1578
BoCD14.84907R	5' GGC AGC CTC TGA GAG TTT ATG T 3'		
			
BoCD14.84746F	5' CTT CCT GTT ATA GCC CCT TTC C 3'	2.5 mM	832
BoCD14.85577R	5' CAC GAT ACG TTA CGG AGA CTG A 3'		
			
BoCD14.85456F	5' GGG TAC TCT CGT CTC AAG GAA C 3'	2.0 mM	825
BoCD14.86280R	5' CTG AGC CAA TTC ATT CCT CTT C 3'		
			
BoCD14.86081F	5' ACC TGA CTC TGG ACG GAA ATC 3'	2.0 mM	747
BoCD14.86827R	5' TAC AGG AGA GCA ACC CTG AAA 3'		

*Primers were designed based on GenBank No. NW_001495367. Numbers preceding F or R in primer names represent their positions on the reference sequence.

PCR reactions with all primer pairs were each carried out in a total volume of 45 μL containing 50 ng DNA, 0.25 mM dNTPs, 2.0 to 2.5 mM MgCl_2 _(Table 3), 10 μM of each primer, 2 units Tag DNA polymerase (Fermentas Life Sciences, Burlington, ON, Canada) and 1× *Taq *buffer. The cycling conditions, with PTC-100™ thermal cycler (MJ Research, Inc., Watertown, MA, USA) included an initial denaturation for 2 min at 94°C followed by 30 cycles comprising 30 sec at 94°C, 30 sec at 60°C, 50 sec at 72°C, and a final elongation for 5 min at 72°C. Both directions of amplified PCR products were sequenced by McGill University/Genome Quebec Innovation Centre using the big dye termination technique and an ABI 3700 sequencer.

### Sequence analysis

Sequences were processed with Chromas, version 1.45 ) and comparison with other published sequences was done with the multiple sequence alignment program with hierarchical clustering, Multalign . CD14 protein sequences of different species (cattle, buffalo, goat, sheep, pig, man, mouse and rat) were aligned or processed with MEGA3.1 software [31] and phylogenetic relationships also constructed with the same software.

### Computational characterization of the bovine CD14 promoter

The promoter region was analyzed for the presence of putative transcription factor-binding sites using the combined search query against the TRANSFAC database with a maximum allowable string mismatch of 10% [[32]; ]. The combined search query option was used to take advantage of the full power of combined string and weight matrix searching, pre-filtering of factors, significance p-values, and new information in new databases. Particular attention was paid to binding sites already proven to be of significance in regulating the CD14 gene of other species and other common binding sites in mouse and human.

### Identification of conserved motifs of regulatory elements in the non-coding regions of the CD14 gene of cattle and other species

Since regions of conserved non-coding sequences between closely related or divergent species are likely to have common functional roles, we searched the region, about 500 bps of the core promoters of cattle (this study or GenBank No. EU148609), human (GenBank No. U00699), mouse (GenBank No. X13987), rat (GenBank No. AF087944) and pig (GenBank No. DQ079063) for described conserved regulatory element (RE) motifs against the NSITEM data base . These species were chosen because of the availability of complete or partial promoter sequence information.

### Flow cytometry

Flow cytometry was used to study the effects of identified CD14 SNPs on the expression of CD14 on the surfaces of neutrophils and monocytes in healthy cows. Blood was collected from the caudal vein of 64 Holstein cows with known CD14 genotypes by venipuncture into vacutainer tubes coated with heparin anticoagulant (BD Biosciences, Franklin Lakes, NJ, USA). After collection, samples were stored on ice and analyzed within four hours. One hundred microlitre of heparinized whole blood was placed in a 12 × 75 mm flow cytometric (FCM) tube and incubated with 10 μL of fluorescein isothiocyanate (FITC)-labeled mouse anti human CD14 antibody (ABD Serotec Inc., Raleigh, NC, USA). This was mixed (Barnstead Thermolyne, Dubuque, IOWA, USA) thoroughly and incubated at room temperature on an orbitron rotator (Boekel Ind. Inc., PA, USA) for 30 minutes. Lysis and fixation of erythrocytes was done by adding 2 mL of lysing solution (PHAGOTEST^® ^Kit, Orpegen Pharma, Heidelberg, Germany) to the mixture. This was mixed gently, incubated for 20 minutes on an orbitron rotator at room temperature and centrifuged at 250 g for 5 minutes at 4°C. The supernatant was aspirated leaving approximately 400 μL of cells in the FCM tube. This was washed with 3 mL of Dulbecco's phosphate buffered saline (DPBS) pH 7.2 (Life Technologies) by centrifuging at 250 g for 5 minutes at 4°C. The supernatant was aspirated as described above and the cells resuspended in 1 mL of DPBS and analyzed by flow cytometry (Becton Dickinson Immunocytochemistry Systems, San José, CA, USA) within 30 minutes. Excitation of samples was at 488 nm; with FITC fluorescence measured at 525 nm ± 10 nm. Acquisition was stopped when 20,000 gated events were collected in the fluorescence cell count histogram. Gating of monocytes and polymorphonuclear leukocytes was based on forward scatter and side scatter dot plots by encircling the populations with amorphous regions. All parameters were recorded with logarithmic amplifications. List mode flow cytometric data from 20,000 events were stored and processed with the Windows Multiple Document Interface for flow cytometry (WinMDI) software version 2.8 (Joseph Trotter, The Sripps Research Institute, ) on a personal computer.

The viability of neutrophils and monocytes in whole blood was determined by propidium iodide (PI) exclusion (50 μg/mL, final concentration) using flow cytometry after cells were incubated for 10 minutes in the dark at room temperature. The cells showed 99% viability.

### Statistical analysis

Allele frequencies were estimated with GENEPOP program [33] while haplotypes and their frequencies were determined with the program PHASE V2.1.1 [34,35]. PHASE implements a Bayesian method of haplotype reconstruction based on genealogies reconstructed from coalescent theory under a Markov Chain Monte Carlo framework and has been shown to outperform other strategies such as the maximum likelihood expectation maximization algorithm in most cases [35].

Flow cytometric data were analyzed as a one-way ANOVA using the MIXED procedure SAS [36]. Treatment means were separated using the least square means option of SAS. Differences between treatment means were tested using Scheffe's Multiple Comparison test and statistical significance was declared at *P *< 0.05.

Statistical model used: Y_ij _= μ + genotype_i _+ e_ij_

## Authors' contributions

EMI-B carried out the molecular genetic studies, sequence and protein comparisons, data analysis and drafted the manuscript. J-WL and XZ conceived the study and participated in its design. XZ attracted funding for the project and coordinated the work. AEI carried out the flow cytometric analysis of cells and analyzed the resulting data. All authors read and approved the draft.
